# TRIM36, a novel androgen-responsive gene, enhances anti-androgen efficacy against prostate cancer by inhibiting MAPK/ERK signaling pathways

**DOI:** 10.1038/s41419-017-0197-y

**Published:** 2018-02-05

**Authors:** Chao Liang, Shangqian Wang, Chao Qin, Meilin Bao, Gong Cheng, Bianjiang Liu, Pengfei Shao, Qiang Lv, Ninghong Song, Lixin Hua, Min Gu, Jie Li, Zengjun Wang

**Affiliations:** 1 Department of Urology, The First Affiliated Hospital of Nanjing Medical University, Nanjing, 210029 China; 2 Department of Pathology, The First Affiliated Hospital of Nanjing Medical University, Nanjing, 210029 China

## Abstract

Hormone therapy drugs, such as bicalutamide and enzalutamide, directed against prostate cancer focus on androgen receptor (AR) signaling and are initially effective, but the disease progresses to lethality as resistance to these drugs develops. A method to prolong the drug response time and improve the drug efficacy is still unavailable. TRIM36 was reported as a novel androgen signaling target gene and is upregulated in prostate cancer. In this study, we found that 63.4% (64/95) of PCa in TMA expressed the TRIM36 protein. Interestingly, patients with negative TRIM36 expression had a shorter biochemical recurrence-free survival. TRIM36 expression was significantly associated with the Gleason score (*P* = 0.005), delayed prostate cancer cell cycle progression and inhibited cell proliferation in vitro and in vivo, and these effects were mediated via inhibition of the MAPK/ERK phosphorylation pathway. Remarkably, we found that rescuing the expression of TRIM36 during anti-androgen therapy could improve the drug efficacy. Collectively, TRIM36 is a novel androgen-responsive gene, and it dramatically enhanced the efficacy of anti-androgen drugs against prostate cancer.

## Introduction

Prostate cancer (PCa) is the most commonly diagnosed cancer in men in the United States and prospectively accounts for 19% of cancer diagnoses in 2017 despite recent rapid declines^1^. Androgen receptor (AR) signaling plays a critical role in tumorigenesis and the development of PCa, and androgen deprivation therapy (ADT) has been used as a standard treatment for patients with advanced prostate cancer^2^. Although ADT is initially effective for patients with hormone-sensitive metastatic prostate cancer, long-term treatment invariably results in the progression to lethal castration-resistant prostate cancer (CRPC)^3^.

The new high-affinity anti-androgen enzalutamide (also known as MDV3100) has been approved recently by the FDA, and it can prolong survival in men with metastatic CRPC^4^. Enzalutamide will once again block AR action in CRPC, and AR action is the key master that influences PCa progression. These findings validate the concept that the AR pathway is a central target for drug therapy^5–9^. Regrettably, modest improvements in survival and rapid resistance to the drugs have limited therapeutic options led to poor prognoses^10,11^.

Historically, testosterone was contraindicated for men with prostate cancer. However, an important paradigm shift has occurred within the field, and testosterone therapy can now be regarded as a viable option for selected men with prostate cancer^12^. Recently, a strategy termed bipolar androgen therapy (BAT), or BAT, has also been found to potentially restore sensitivity to ADT^13^. Therefore, it is necessary to review the dual function of AR signaling target genes, which may aid the discovery of the mechanism of drug resistance and lead to new therapeutics to enhance the efficacy of anti-androgen therapy.

With ChIP-chip and CAGE (cap analysis of gene expression) analysis, which are advanced high-throughput techniques, several new AR target genes, including TRIM36, have been identified in the whole genome of prostate cancer cells^14–16^. A high level of TRIM36 expression has been observed in PCa tissues^17^. In our previous study, TRIM36 was also found to be highly expressed in primary PCa^18^. TRIM36 is a novel E3 ubiquitin ligase that interacts with the kinetochore protein CENP-H and decelerates the cell cycle progression of NIH3T3 cells^19^. Our present studies aim to investigate the functions of TRIM36 in PCa and how to enhance the efficacy of anti-androgen therapy.

## Materials and methods

### Cell culture and specimens

The RWPE-1, C4–2, LNCAP, DU145, and PC-3 human prostatic cancer cell lines were obtained from the Cell Bank Type Culture Collection. The RWPE-1 cells were cultured in keratinocyte serum-free medium (K-SFM) (Gibco, USA) with bovine pituitary extract (BPE) and human recombinant epidermal growth factor (EGF). The PC-3 and DU145 cells were cultured in F-12K Nutrient Mixture (Gibco, USA), and the C4–2 and LNCAP cells were cultured in RPMI-1640 (Gibco, USA). All media were supplemented with 10% fetal bovine serum (FBS, Gibco, USA) and cells were maintained in a humidified atmosphere containing 5% CO_2_ at 37 °C.

Twenty-four PCa tissues were included in this study for mRNA extraction. The tissues were obtained from patients who underwent radical prostatectomy at the Department of Urology of the First Affiliated Hospital of Nanjing Medical University. The specimens were snap frozen in liquid nitrogen after surgery and stored at −80 °C until use.

### Patients and tissue micro arrays (TMAs)

The PCa patients used for the creation of TMAs in this study have been described previously^20^. Briefly, 95 prostate cancer tissues were obtained from patients who were treated by radical prostatectomy between 2008 and 2011 at the First Affiliated Hospital of Nanjing Medical University (Nanjing, China). All patients were recruited following the acquisition of informed consent. For the present study, the clinical and pathologic features of all patients are summarized in

**Table 1:** Relationship of TRIM36 expression and clinicopathologic characteristics of patients

Variable	TRIM36 expression		
	Negative (*n* = 31)	Positive (*n* = 64)	*P* value
Age			0.605
<60	3	3	
60–70	14	28	
>70	14	33	
Preoperative PSA (ng/ml)			0.649
<10	7	20	
10–20	10	20	
>20	14	24	
Gleason score			**0.005**
≦6 or =3+4	12	44	
=4+3 or ≧8	19	20	
T stage			0.950
pT2	26	54	
pT3/ T4	5	10	
Biochemical recurrence			**0.001**
Negative	12	48	
Positive	19	16	

Bold values signify *P* < 0.05. *P* values were two-tailed and based on the Pearson chi-square test

Table 1. Biochemical recurrence (BCR) was defined as two consecutive postoperative increases in prostate-specific antigen (PSA) of 0.2 ng/ml or greater in the serum. The follow-up deadline was 30 April 2016. The protocols used in the study were approved by the ethics committee of the hospital. This study was approved by the medical ethics committee of the hospital.

### Immunohistochemistry (IHC) and evaluation of the staining

IHC staining was performed as previously described^21^. Ninety-five prostate cancer samples were analyzed. The primary antibodies were incubated as follows: anti-TRIM36 (SAB2106623, 1:200, Sigma) and anti-AR (ab74272, 1:200, Abcam). The evaluation of protein staining was separately and independently performed by two experienced pathologists without knowledge of the clinical data. The results of IHC staining for TRIM36 were determined by the Amend Allred scoring system as described in previous studies^21,22^. Briefly, the percentage of positive tumor cells was determined in at least five areas at 400× magnification and assigned to one of the following five categories: 0, <5%; 1, 5–25%; 2, 25–50%; 3, 50–75%; and 4, >75%^23^. The intensity of the immunostaining was scored as follows: 1, low; 2, moderate; and 3, strong. Given the homogeneity of the staining of the target proteins, the predominant pattern was taken into account for scoring. The IHC scores of the PCa tissues for TRIM36 were as follows: negative expression (<1) and positive expression (1–12). The scores for AR were as follows: low expression (<4) and high expression (5–12).

### Cell transfection

Two lentiviral vectors with TRIM36 shRNA and one that over-expressed TRIM36 were constructed by Genechem (Shanghai, China). A lentiviral vector with NC shRNA was used as a negative control for the TRIM36 knockdown. The lentiviral vector was used as a negative control for TRIM36 over-expression.

Cells of the LNCAP, C4–2, and PC-3 lines were seeded in 6-well plates at 40% confluence on the day before transfection. The lentivirus that containing TRIM36 expression vector or empty vector alone was used to infect LNCAP, C4–2, and PC-3 cells at a multiplicity of infection (MOI) of 20. Three days after infection, GFP expression was detected to assess the infection efficiency. Five days after infection, the cells were harvested and split into two parts. Real-time reverse transcription polymerase chain reaction (RT-PCR) and Western blotting were performed to evaluate TRIM36 expression efficiency in one part of cells. Cells with TRIM36 knocked down were defined as TRIM36-Sh1 and TRIM36-Sh2, those over-expressing TRIM36 were defined as the TRIM36-OV group, and the cells that were transfected with lentiviral vector alone were defined as the NC group.

### RNA isolation and RT-PCR

After treatment, mRNA was isolated using Trizol reagent according to the manufacturer’s instructions (Invitrogen). The cDNA was prepared using the High Capacity Reverse Transcription kit from Applied Biosystems (Foster City, CA). The RT-PCR primers were synthesized at Midland Certified Reagent Company (Midland, TX), and SyBr Green Master Mix was purchased from Applied Biosystems (Foster City, CA). The following primer sequences were used for quantitative RT-PCR: beta actin: 5′-TCCCATCACCATCTTCCA-3′ and 5′-CATCACGCCACAGTTTCC-3′; and TRIM36: 5′-GAGCTGTTTACCCACCCATTG-3′ and 5′-CTGATCCCACATCGTTGAATGA-3′. Fold changes in gene expression were calculated after normalization to their corresponding beta actin mRNA levels.

### Plasmids construction

The lentiviral vector with overexpression of TRIM36 was constructed by Genechem (Shanghai, China). The lentiviral vector alone was used as a negative control for transfection.

TRIM36 promoter containing first exon (1563 bps) and TRIM36 promoter containing first exon and Δintron which includes the intronic region containing androgen receptor binding sites (ARBS) (1722 bps) were inserted between the KpnI and XhoI sites of pGL3 promoter vectors (Genscript, Nanjing, China). The accuracy of the constructed plasmids was verified by DNA sequencing.

### Dual-luciferase assay

HEK-293 and LNCAP cells were used for cell transfection and luciferase assays. The cells were seeded into culture medium-containing (100 μL/well) 96-well plates at a cell concentration of 1.5 × 104 cells/well, followed by a 24-h incubation (37 °C, 100% humidity, and 5% CO_2_). The cells were allocated into two groups: Group A was cultured with charcoal stripped fetal bovine serum (CSS), and Group B was cultured with Metribolone (R1881) (10 nM) in medium. In each group, there were two sub-groups: Sub-group A was transfected with TRIM36 promoter, and Sub-group B was transfected with TRIM36 promoter + Δintron using Lipofectamine 2000 (Invitrogen Corp, CA, USA). As an internal standard, all plasmids were cotransfected with pRL-SV40, which contained the Renilla luciferase gene. After transfection for 48 h, luciferase activity was measured with a Dual-Luciferase Reporter Assay System (Promega). Independent triplicate experiments were performed for each plasmid construct.

### Western blot analysis

The cells were lysed in RIPA buffer, and the proteins (20 μg) were separated on 10% SDS/PAGE gels and then transferred onto PVDF membranes (Millipore, Billerica, MA, USA). After blocking the membranes with 5% non-fat milk, they were incubated with the appropriate dilutions of specific primary antibodies (1:1000). The blots were then incubated with HRP-conjugated secondary antibodies (1:4000). Antibodies against TRIM36 (Sigma, GER), glyceraldehyde 3-phosphate dehydrogenase (GAPDH; Bioworld Technology, USA), extracellular signal-regulated kinase (ERK), p-ERK, p-MSK1, c-myc, cyclin D1, and cyclin E1 (Cell Signaling Technology, USA) were used in Western blot analysis in accordance with the manufacturer’s instructions. The blots were detected using enhanced chemiluminescence (Thermo Scientific). The protein levels were determined by normalization to GAPDH.

### Cell cycle analysis

The cell cycle distribution was analyzed by flow cytometry (Becton Dickinson). The cells were harvested, washed twice with ice-cold phosphate-buffered saline and fixed with 70% ethanol for at least 12 h at −20 °C. The fixed cells were incubated in 50 mg/ml of propidium iodide and 1 mg/ml of RNase for 30 min at room temperature. At least 20,000 cells were acquired for each sample. The experiments were performed in triplicate.

### Clone formation assay

The 6-well plates were seeded with 1000 LNCAP or 500 PC-3 cells per well. The LNCAP cells were transfected with TRIM36-ShRNAs or NC-ShRNA. The PC-3 cells were transfected with the lentiviral vector with TRIM36 or NC. After 2 week, the cells were stained with 0.1% crystal violet.

### Xenograft

The mouse studies were approved by the Animal Research Ethics Committee of Nanjing Medical University. (1) flank implantation: five-week-old male nude mice were randomly divided into two groups consisting of five mice each. The stable Lv-TRIM36-PC-3 cells (5 × 10^6^) and the control cells (NC-PC-3) were suspended in 150 μl PBS and injected subcutaneously into the flank of each mouse. Tumor size was calculated (length × width^2^ × 0.52) once per week. After 6 weeks, the tumors were removed, weighed, and fixed. (2) Prostate orthotopic implantation: The Matrigel mixtures with 5 × 10^6^ C4-2 cells (with stable transfected GFP) with or without expression of TRIM36 were orthotopically injected into both anterior prostates. After one week implantation, the mice were randomly assigned into four experimental groups (injection of C4-2-GFP cells with or without expression of TRIM36 and treated with either DMSO or 35 mg/kg Enz by i.p. injection 3x/week for 2 weeks). Tumor formation and sizes were monitored weekly during the 2 weeks of drug treatment. After another week and a final imaging by fluorescence microscopy, mice were sacrificed and tumors were removed. The tissue samples were then fixed and processed as paraffin tissue sections.

### Chromatin immunoprecipitation (ChIP) assay

For the ChIP assays, we used the EZ ChIP™ Chromatin Immunoprecipitation Kit (Catalog # 17-371, Millipore). The assays were performed according to the manufacturer’s protocols with minor modifications. Briefly, R1881-treated (6 h) LNCAP cells were sheared using the Covaris truChIP kit using an E220 focused-ultrasonicator from Covaris. The chromatin samples were diluted in ChIP buffer from the Active Motif kit. The samples were then immunoprecipitated (IP) with the AR antibody (Abcam ab74272). The remainder of the assay was performed according to the Active Motif kit instructions. ARE-specific RT-PCR was performed on the IP DNA using primers located within TRIM36 intron (5′-CAATGGCAGATATCACTGTGTACTTTAAA-3′, and’-GTATGTTCTCTGAAATGTGGGAAGTAAG-3′).

### Cell proliferation assay

Different pretreated cells were seeded into 96-well plates at a density of 1.5 × 10^3^ cells/well and cultured for 24, 48, 72, or 96 h. Cell proliferation was assayed using a Cell Counting Kit-8 (CCK-8; Dojindo Molecular Technologies, Japan) in accordance with the manufacturer’s protocol. Absorbance was detected at the wavelength of 450 nm. Three wells were measured for cell viability in each group.

### Statistical analyses

All statistical analyses were performed with SPSS 16.0 (SPSS Inc., Chicago, IL, USA).

We used the Pearson correlation method to analyze the relationship between TRIM36 expression and clinicopathological factors. Univariate BCR-free survival was assessed using Kaplan–Meier curves and log-rank tests. A Cox proportional hazards regression model was used to identify the univariate and multivariate hazard ratios for the variables of this study. The results are expressed as the means ± the standard deviations (SDs). In vitro differences between groups were subjected to Student’s *t*-tests. *P* < 0.05 was considered statistically significant.

## Results

### Co-relationship between clinical factors and the expression of TRIM36 in prostate cancer

**Fig. 1:**
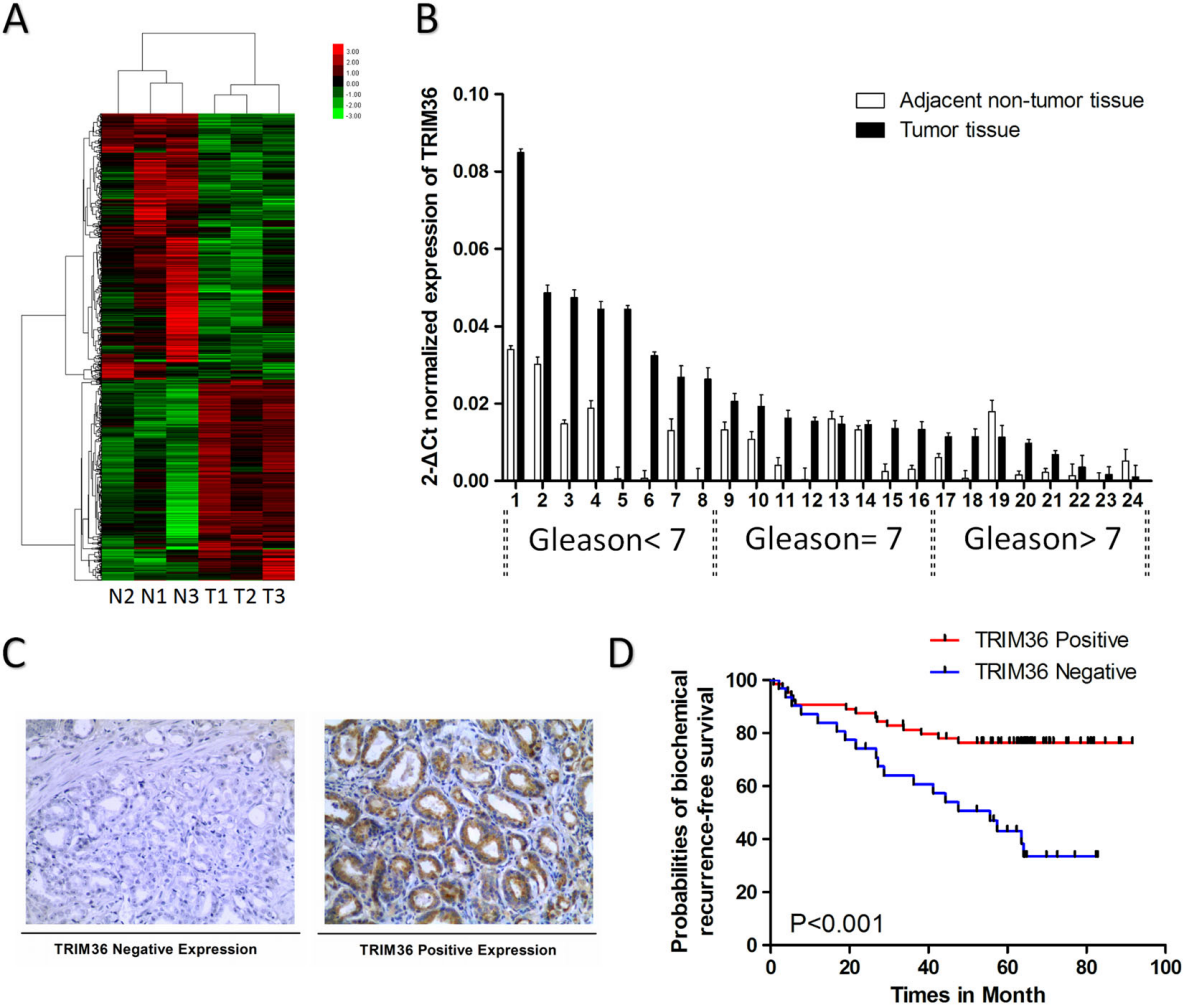
Co-relationship between clinical factors and the expression of TRIM36 in prostate cancer. **a** TRIM36 was upregulated in our mRNA microarray data. **b** Relative mRNA expression of TRM36 in PCa tissues with different Gleason scores compared with the corresponding non-tumor tissues. The TRIM36 mRNA level is higher in the PCa tissues with low Gleason scores. **c** Different immunohistochemistry results for TRIM36 expression in a tissue microarray. Left: Negative TRIM36 expression. Right: Strong TRIM36 expression. **d** Kaplan–Meier biochemical recurrence-free survival curves for PCa patients based on TRIM36 expression levels. Patients with positive TRIM36 expression had obviously longer survival times than those with negative TRIM36 expression (log-rank test, *P* < 0.001)

TRIM36 over-expression has been observed in PCa tissues^18^, and this observation was confirmed in our previous microarray data (Fig. 1a). TRIM36 mRNA expression was analyzed in 24 PCa samples by RT-PCR (Fig. 1b). The expression of TRIM36 was significantly higher in the PCa tissues compared with the adjacent non-tumor tissues. However, TRIM36 expression in high Gleason score samples was found to be lower than that in low Gleason score samples.

To analyze the function of TRIM36 in PCa, we determined TRIM36 protein expression in 95 cases of prostate cancer (Fig. 1c). Immunostaining revealed that the percentage of TRIM36 positivity was 63.4% (64/95) in the PCa samples. The relationships of TRIM36 expression and the clinicopathologic characteristics of patients are listed in Table 1. There were no significant differences in TRIM36 expression with age, preoperative PSA or tumor stage (*P* > 0.05). However, TRIM36 expression was significantly negatively associated with Gleason score (GS) (*P* = 0.005) and BCR (*P* = 0.001). Univariate Kaplan–Meier/log-rank analysis also indicated that negative TRIM36 protein expression was significantly related to an increased risk for poor clinical outcome in PCa patients (log rank *P* < 0.001, Fig. 1d). The patients with negative TRIM36 expression had shorter disease-free survival.

Together, the results from Fig. 1a–d suggest that TRIM36 expression was upregulated in prostate cancer but negatively associated with poor prognosis.

### Effects of TRIM36 on prostate cancer cell cycle and proliferation

**Fig. 2:**
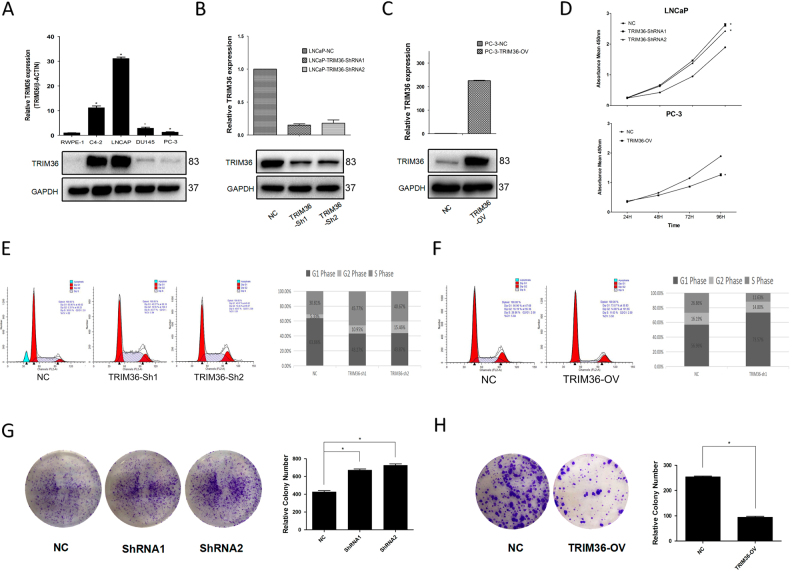
Function of TRIM36 in PCa cell lines. **a** Expression of TRIM36 in prostate cancer cell lines. Top: Relative mRNA expressions of TRIM36 in PCa cell lines (C4–2, LNCAP, DU145, and PC-3) and the normal human prostate epithelial cell line RWPE-1. Bottom: Relative protein expression of TRIM36 in PCa cell lines (C4–2, LNCAP, DU145 and PC-3) and RWPE-1. **b**, **c** The knockdown and overexpression of TRIM36 were confirmed. **b** The knockdown and overexpression of TRIM36 in LNCAP and PC-3 cell lines were confirmed with RT-PCR to detect the relative mRNA expression of TRIM36 (*P < 0.05). **c** The knockdown and overexpression of TRIM36 in LNCAP and PC-3 cell lines were confirmed with western blot to detect the protein expression of TRIM36 (*P < 0.05). **d**–**h** TRIM36 regulates cell cycles and proliferation. **d** The knockdown of TRIM36 significantly promoted cell growth in the LNCAP line, while the overexpression of TRIM36 significantly inhibited cell growth in the PC-3 line. **e**–**f** The knockdown of TRIM36 prevented G0/G1 phase cell cycle arrest in the LNCAP line, while the overexpression of TRIM36 resulted in G0/G1 phase cell cycle arrest in the PC-3 line. **g**, **h** The knockdown of TRIM36 obviously increased colony formation efficiency in the LNCAP line, while the overexpression of TRIM36 decreased colony formation efficiency in the PC-3 line

We selected five cell lines, i.e., RWPE-1, C4–2, LNCAP, DU145, and PC-3, to investigate their TRIM36 expression levels as determined by RT-PCR and Western blot. High levels of the expression of TRIM36 were observed in the AR-positive prostate cancer cell lines (LNCAP and C4-2), whereas RWPE-1 cells rarely exhibited expression of TRIM36 (Fig. 2a).

Because recent clinical data indicated that TRIM36 expression might decelerate the cell cycle progression of NIH3T3 cells^20^, we were interested to determine whether TRIM36 might alter prostate cancer cell cycle and proliferation. According to the TRIM36 expression in each cell line, the LNCAP line was chosen for the subsequent knockdown experiments, and the PC-3 line was selected for the overexpression experiments. The stably decreased TRIM36 levels in the LNCAP line and the over-expression in the PC-3 line were confirmed by RT-PCR and Western blot analyses (Fig. 2b, c).

We first applied the flow cytometry to examine the percentages of cells in the G0/G1, S, and G2/M phases. As illustrated in Fig. 2e–f, the knockdown of TRIM36 reduced the number of the LNCAP cells in the G1 phase, and the opposite results were obtained after exogenous TRIM36 expression in the PC-3 cells.

We then applied different cell growth/viability assays and clonogenic formation assays to further confirm the effects of TRIM36 in the LNCAP and PC-3 cells. The results revealed that TRIM36 suppressed cell growth in the LNCAP and PC-3 cells (Fig. 2d, g, h).

**Fig. 3:**
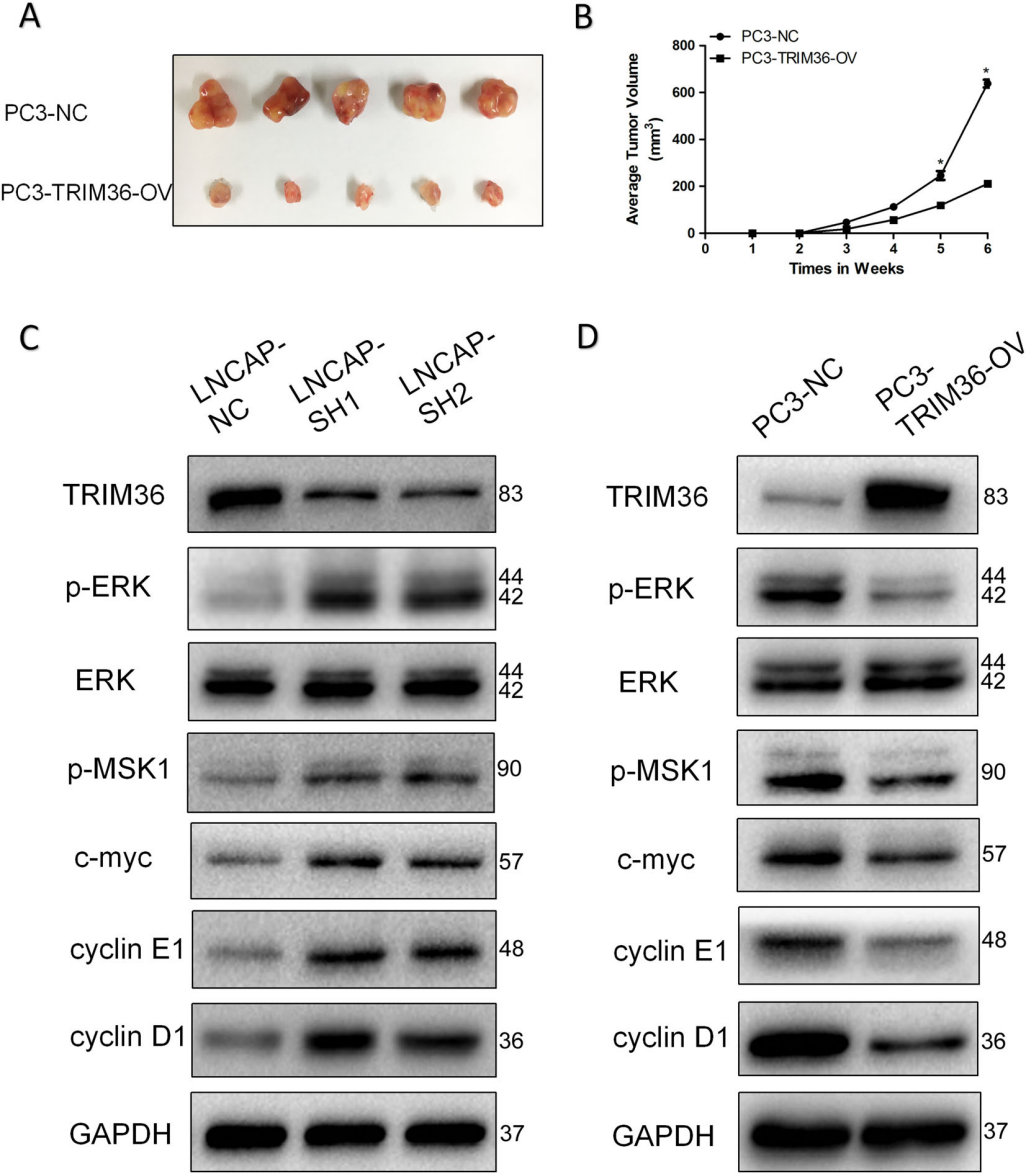
TRIM36 inhibits the MAPK/ERK phosphorylation pathway. **a**, **b** TRIM36 significantly affected cellular growth in vivo. **a** Representative pictures of tumors. **b** Tumor volumes were measured at the indicated number of days after the mice were injected with tumor cells. Each bar represents the mean tumor volume ± the S.D. of five mice per group. *P < 0.05. **c** The knockdown of TRIM36 promoted changes in MAPK/ERK phosphorylation pathway marker expressions with gains in p-ERK, p-MSK1, C-MYC and Cyclin E1, and Cyclin D1 in the LNCAP line. **d** In contrast, the overexpression of METTL3 promoted changes with losses in p-ERK, p-MSK1, C-MYC and Cyclin E1, and Cyclin D1 in the PC-3 line. GAPDH was used as a loading control. *P < 0.05

As shown in Fig. 3a, tumors derived from PC-3 cells with overexpression of TRIM36 grew much slower than those derived from PC-3 NC cells, which were consistent with in vitro results (Fig. 3b). The results demonstrated that overexpression of TRIM36 significantly inhibited tumor growth.

Together, the results from Figs. 2a–h and 3a, b suggested that TRIM36 could delay the cell cycle and suppress the proliferation of prostate cancer cells in vitro and in vivo.

### TRIM36 inhibits the MAPK/ERK phosphorylation pathway

To further study the mechanism by which TRIM36 overexpression or knockdown affected the cell cycle and proliferation, we investigated the effects of TRIM36 overexpression or knockdown on the MAPK/ERK pathway. Western blot analyses suggested that the levels of phospho-MAPK/ERK, phospho-MSK1, and c-myc increased in the TRIM36 knockdown cells, while these levels decreased in the TRIM36 over-expressing cells. However, the total levels of MAPK/ERK exhibited no obvious changes in the TRIM36 overexpressing or knockdown cells. Moreover, the G1/S phase markers Cyclin D1 and Cyclin E1 increased in the TRIM36 knockdown cells, while these levels decreased in the TRIM36 over-expressing cells (Fig. 3c, d).

### Androgen-responsive expression of TRIM36 in prostate cancer cells

**Fig. 4:**
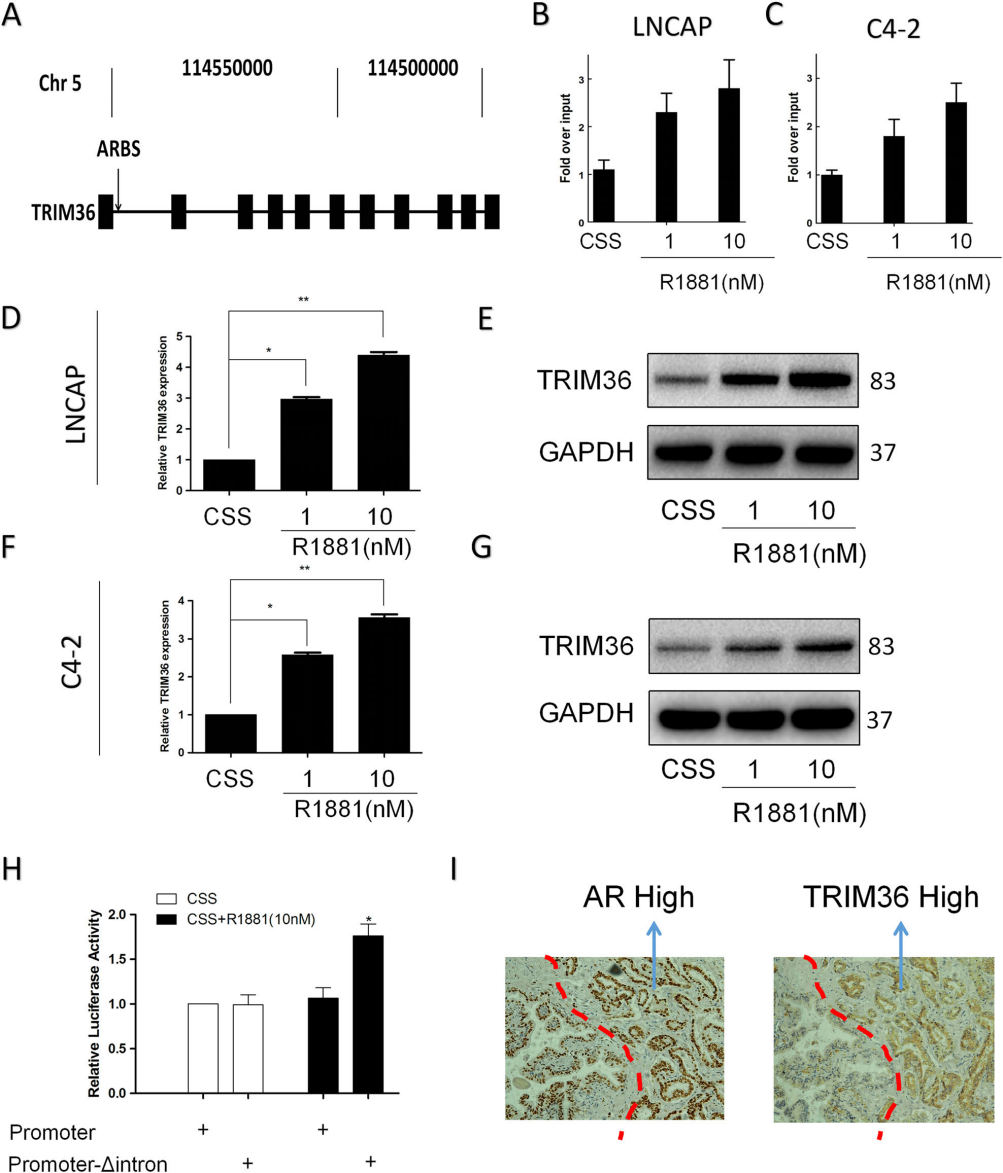
Expression of TRIM36 is upregulated by androgen stimulation in LNCAP cells. **a** Genomic view of the TRIM36 gene in the UCSC genome browser. ChIP-chip analysis identified an ARBS in the first intron region of TRIM36^16^. **b**–**c** Validation of ligand-dependent androgen receptor recruitment to the TRIM36 ARBS using a ChIP assay. LNCAP (**b**) and C4–2 (**c**) cells were treated with R1881 (1/10 nM) or CSS for 24 h. **d**–**g** Induction of TRIM36 by androgen treatment in LNCAP cells. **d**,** f** RT-PCR revealing androgen-dependent upregulation of TRIM36 mRNA in LNCAP and C4–2 cells. LNCAP cells were treated with R1881 (1/10 nM) or CSS. The TRIM36 mRNA levels are plotted relative to those of the CSS control. **e**,** g** Androgen-mediated induction of TRIM36 protein expression in LNCAP and C4–2 cells. Protein levels were analyzed by Western blot analysis. GAPDH was used as a loading control. TRIM36 protein levels were quantified by densitometry and normalized to GAPDH levels. **h** LNCAP cells were transfected with TRIM36-promoter reporter plasmid or TRIM36-promoter + Δintron reporter plasmid, and treated with R1881 (10 nM) or CSS for 24 h. TRIM36 promoter activity was significantly increased in the TRIM36 promoter + Δintron group with R1881 (10 nM) in a dual-luciferase assay. **i** The TRIM36 gene is overexpressed in prostate cancer tissues with the same pattern of AR expression in the nucleus

An integrative analysis was previously reported that used ChIP-chip and CAGE to reveal that TRIM36 includes an intronic ARBS^17^ (Fig. 4a). IHC of the prostate cancer tissues revealed the same pattern of AR and TRIM36 staining (Fig. 4i). These results indicate that TRIM36 may be regulated by androgens in prostate cancer.

First, we validated the Dihydrotestosterone (DHT)-dependent AR recruitment to the ARBS using a ChIP assay in the LNCAP and C4-2 cells (Fig. 4b, c). Next, to investigate the effect of androgen on the regulation of TRIM36 mRNA expression, we stimulated LNCAP and C4-2 cells with R1881 (1, 10 nmol/L) or control vehicle, and RT-PCR analysis revealed that TRIM36 mRNA levels were increased by androgen stimulation relative to control treatment (Fig. 4d, f). Western blot analysis also revealed that TRIM36 protein expression was upregulated by androgen stimulation (Fig. 4e, g). Additionally, a dual-luciferase assay indicated that the TRIM36 promoter activity was significantly increased in the TRIM36 promoter + Δintron cells with R1881 (10 nmol/L) stimulation (Fig. 4h).

Together, these results in Fig. 4a–i suggest that TRIM36 is an androgen-responsive gene that is regulated by AR binding.

### Anti-androgen therapy reduces TRIM36 expression

**Fig. 5:**
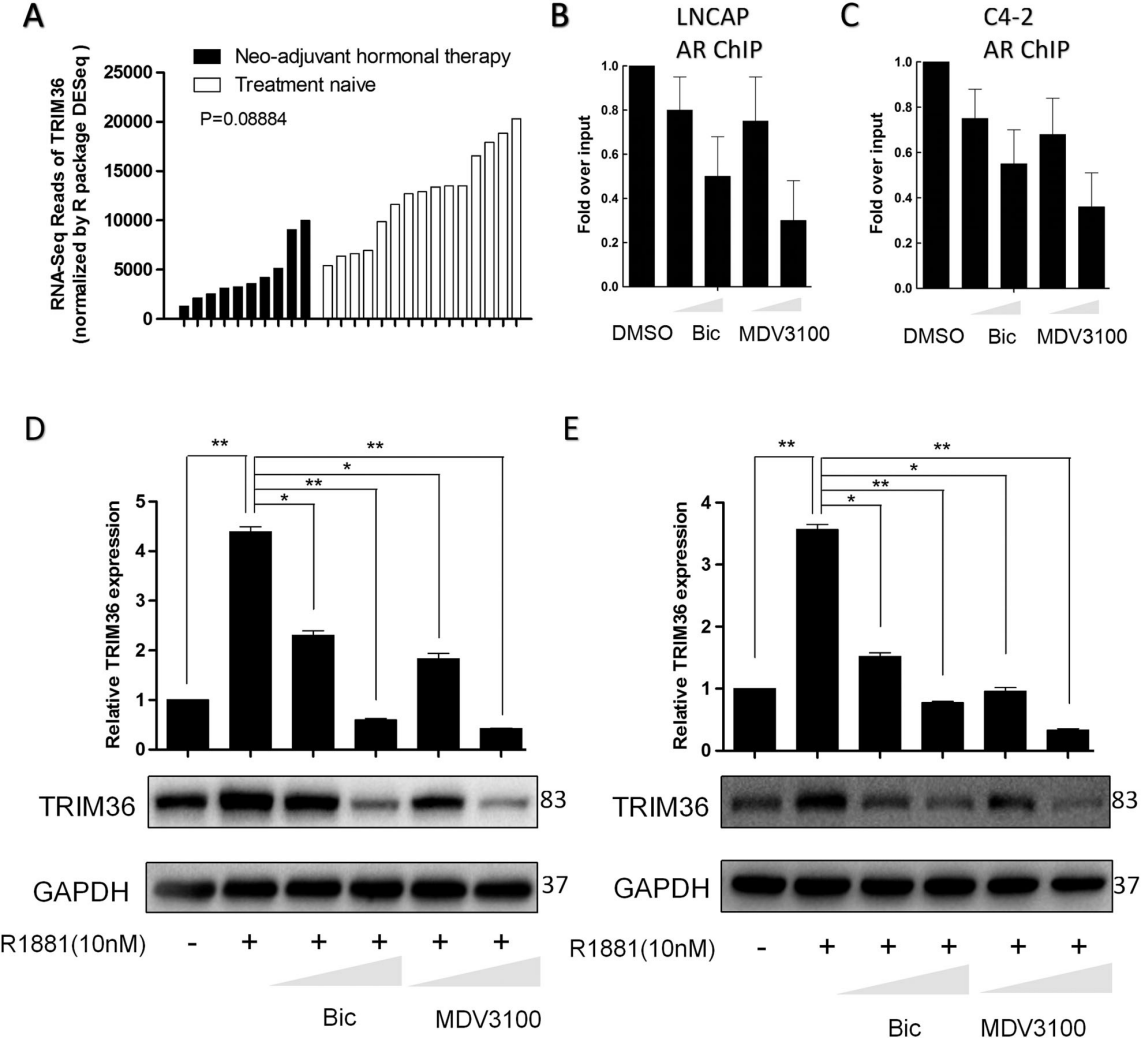
Anti-androgen therapy reduces TRIM36 expression. **a** RNA-seq database showing the TRIM36 expression reduced in the ten PCa tumors that were exposed to neoadjuvant hormone therapy compared with treatment-naive samples. **b**, **c** Bicalutamide and MDV3100 inhibit AR recruitment to the TRIM36 ARBS based on a ChIP assay. LNCAP and C4–2 cells were treated with R1881 (10 nM), R1881 + bicalutamide (Bic; 1 or 10 μM), or R1881 + MDV3100 (MDV; 1 or 10 μM) for 24 h. **d**, **e** Bicalutamide and MDV3100 inhibit the androgen-mediated upregulation of TRIM36. LNCAP and C4–2 cells were treated with CSS or R1881 (10 nM), R1881 + bicalutamide (Bic; 1 or 10 μM), or R1881 + MDV3100 (MDV; 1 or 10 μM) for 24 h, *P* < 0.01

Ten high-risk PCa tumors exposed to neo-adjuvant hormone therapy exhibited reduced TRIM36 expression compared with that in the treatment-naive samples^24^ (Fig. 5a). In this study, we found that the AR inhibitors bicalutamide and MDV3100 reduced AR recruitment to the ARBS using a ChIP assay in the LNCAP and C4-2 cells (Fig. 5b, c). Then, we stimulated LNCAP and C4-2 cells with bicalutamide or MDV3100 (1, 10 μmol/L) or control vehicle, and RT-PCR analysis and western blot analyses revealed that TRIM36 mRNA and protein levels were decreased by AR inhibitors relative to the control treatment (Fig. 5d, e). The DHT-induced upregulation of TRIM36 was inhibited by treatment with bicalutamide or MDV3100.

### Rescued TRIM36 increased the anti-androgen sensitivity

**Fig. 6:**
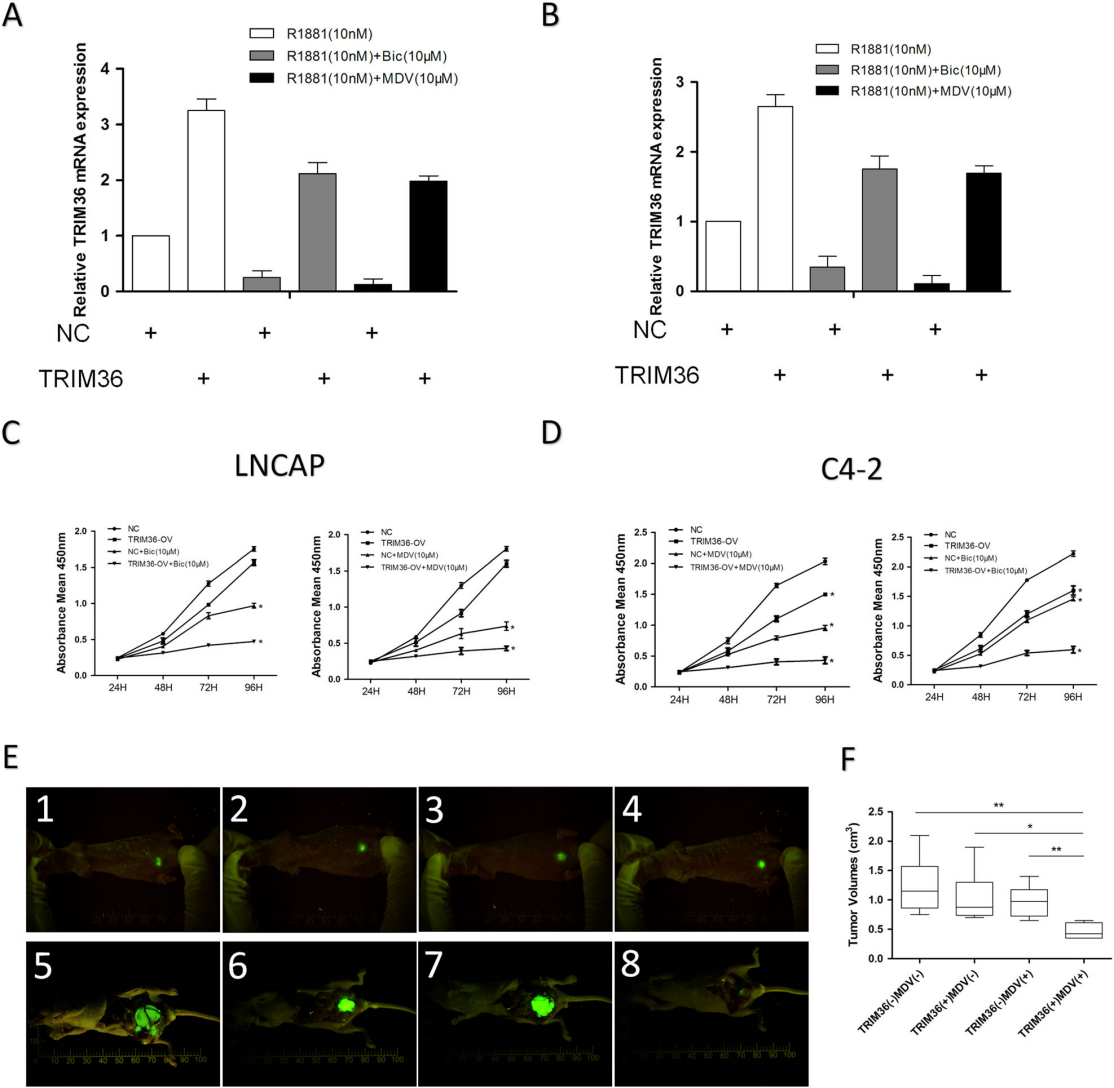
Rescued TRIM36 increased the anti-androgen sensitivity in vitro and in vivo. **a**, **b** Validation of rescued TRIM36 expression after anti-androgen drugs in LNCAP and C4–2 cell. **c**, **d** The rescued TRIM36 alters the anti-androgen drugs efficacy by inhibiting prostate cancer cell growth. **e** C4–2 cells with or without expression of TRIM36 were labeled with GFP and injected orthotopically into nude mice. Two week after implantation, tumors were formed and visualized by fluorescence image. Representative fluorescence images of each group are shown (**e1**–**e4**). Sequential in vivo whole body fluorescence imaging of tumor progression in different groups. After another 3 weeks and a final fluorescence imaging, mice were sacrificed and tumors were removed (**e5**–**e8**). **f** Tumor sizes in the four groups after sacrifice and tumor removal. *P < 0.05, **P < 0.01

We then applied different cell growth/viability assays to further confirm the effects of TRIM36 and the AR inhibitors on the LNCAP and C4-2 cells, and the results revealed that adding TRIM36 and AR inhibitors led to the suppression of cell growth in the LNCAP (Fig. 6a, c) and C4-2 lines (Fig. 6b, d). To test the validity of all above in vitro cell line results in the preclinical study using in vivo mouse model, we orthotopically xenografted the C4-2 cells (with stable expression of GFP) into the anterior prostates of nude mice for 4 groups: (1) C4-2-GFP alone, (2) C4-2-GFP over-expressing TRIM36, (3) C4-2-GFP + treated with MDV3100, (4) C4-2-GFP over-expressing TRIM36 + treated with MDV3100. One week after orthotopic injection, tumor formations were visualized weekly by imaging (Fig. 6e). The baseline tumor sizes of each group were roughly similar by fluorescence microscopy. In general, tumor growth in vivo matched cell growth in vitro showing MDV3100 treatment for two weeks suppressed the C4-2-GFP tumor growth (group 3 vs group 1; 21% decrease in tumor size) compared to C4-2-GFP tumor growth, although without a statistical significance. Expressing TRIM36 alone also suppressed the C4-2-GFP tumor growth (group 2 vs group 1; 17% decrease in tumor size). Importantly, combining MDV3100 and TRIM36 led to the most significant suppression effects (group 4 vs group 1; 62% decrease in tumor size; Fig. 6f).

Importantly, we found that this suppression of prostate cancer cell growth could be enhanced after reversing the expression of TRIM36 in vitro and in vivo, which suggested that TRIM36 may increase the anti-androgen sensitivity in prostate cancer.

## Discussion

The impressive effect of ADT with anti-androgen treatments to prevent androgens from binding to the AR identified by Huggins and Hodges in 1941 was of paramount significance in prostate cancer^2^. However, after an initial effective response to ADT therapy, eventually, PCa will develop into castration-resistant PCa (CRPC)^24,25^. Although neoadjuvant hormone therapy represents breakthroughs in the treatment of metastatic CRPC, approximately 40% of patients have no response to these agents with respect to PSA levels^26,27^. PCa can acquire adaptive autoregulation to maximal androgen ablation or AR signaling pathway blocking.

Interestingly, some AR-expressing prostate cancer cells can be inhibited by exposure to supraphysiologic androgen levels^28^, and BAT may restore CRPC cells to androgen sensitivity and thus sensitivity to traditional ADT^13^. These data are thought provoking in light of the dramatic shift in our understanding of the role androgens and AR play in prostate cancer. Therefore, it is necessary to review the functions of AR and AR signaling target genes to investigate the possible explanations behind this phenomenon. As a member of the nuclear receptors, AR functions as a ligand-activated transcriptional factor that regulates downstream target genes to alter various cell functions. Takayama et al. used the high-throughput CAGE method to map androgen-regulated promoters in the human genome^16^. TRIM36 was identified and includes an intronic CAGE TC within the second AcH3 site that is situated downstream of the intronic ARBS. Early pilot data from the same investigators suggest that the expression of TRIM36 in cancer cells from men without PSA recurrence is significantly increased, and this condition was identified as a prognostic factor for cancer-specific survival^29^. Here, we analyzed the expression of TRIM36 in specimens obtained from 95 PCa samples. The results revealed that the TRIM36 protein was expressed in 64 of all of the PCa patients, and this result was confirmed by LC-MS/MS (Supplement Fig. 1). Furthermore, the patients with negative TRIM36 expression had a shorter BCR-free survival than those who were positive for TRIM36 expression. Our results strongly suggest that negative TRIM36 expression could be of clinical value as a prognostic indicator of BCR.

TRIM36 is a member of the TRIM family that was cloned from the tumor suppressor gene region located at chromosome 5. It has been reported that the TRIM36 protein interacts with centromere protein-H and that the over-expression of TRIM36 results in cell cycle arrest^20^. Moreover, the expression of TRIM36 is significantly down-regulated in several NSCLC cell lines^30^. However, TRIM36 has been reported to be upregulated in primary prostate cancer, and this result was also confirmed in our PCa cohort. The enhanced expression of TRIM36 due to AR signaling in prostate cancer is considered to be a reasonable explanation for this phenomenon. Consistent with clinical data, we found that TRIM36 was expressed in the AR-positive cell lines LNCAP and C4-2, while in the AR-negative PC-3 and DU145 lines, TRIM36 was barely detectable. Further knockdown and over-expression experiments also demonstrated that TRIM36 markedly reduced the cell growth rate and delayed the cell cycle. Additionally, xenograft studies revealed TRIM36-upregulated PC-3 cells formed smaller tumors compared to the control cells. Recent studies have revealed that hypermethylation of TRIM36 appears in endometriosis and associated ovarian carcinomas and neuroblastoma tumors, which indicates that TRIM36 hypermethylation might be involved in cancer development^31,32^. We performed a bisulfite sequencing PCR (BSP) assay on DNA samples isolated from primary PCa tissues with different Gleason scores. As expected, the TRIM36 methylation level increased in the high GS tissues (Supplement Fig. 1D). This finding might explain the low TRIM36 expression in the high-GS PCa tissues compared with the low-GS tissues. Moreover, these data suggested that TRIM36 acted as a tumor suppressor during PCa progression.

Inappropriate cell survival and uncontrolled proliferation are among the characteristics of cancer, and these processes are commonly regulated by the MAPK/ERK pathway^33–35^. Inversely, we clearly demonstrated here that TRIM36 regulated the activity of the MAPK/ERK/MSK-1 pathway as represented by the phosphorylation wave of this signaling. The MAPK/ERK signaling pathway plays an important role in the regulation of prostate cancer cell functions, including metabolism, proliferation, protein synthesis, and survival^36,37^. It was reported that there is a positive feedback loop between the AR and ERK signaling pathways. Moderate DHT could promote cancer cell proliferation with increasing ERK phosphorylation^38^. For the first time, the present study elucidated the mechanism of the regulation of MAPK/ERK/MSK-1 by TRIM36. The mechanism may be the dual-suppression of ERK signaling by exogenous overexpression of TRIM36 and ADT.

Our study also dissected the mechanism of androgen-influenced TRIM36 expression and found that the anti-androgen drugs bicalutamide and MDV3100 could suppress the TRIM36 level. The TRIM36 promoter activity can be enhanced by the stimulated AR signaling, which can also be reversed by anti-androgen drugs. In this study, we applied cell growth/viability assays to confirm the AR inhibitors’ effects in the AR-positive prostate cells and that the inhibition of cell growth could be enhanced by rescuing TRIM36 expression.

In conclusion, we found that TRIM36 is a novel androgen-responsive gene that enhanced the efficacy of anti-androgen drugs against prostate cancer. Addition of TRIM36 during ADT therapy may be developed as a new therapeutic approach to better suppress CRPC.

## Electronic supplementary material

Figure S1

Table S1
