# Supplementary figures and images for: TRIM36, a novel androgen-responsive gene, enhances anti-androgen efficacy against prostate cancer by inhibiting MAPK/ERK signaling pathways
Source: Cell Death Dis. 2018 Feb 5;9(2):155. doi: 10.1038/s41419-017-0197-y (PMC5833828; doi:10.1038/s41419-017-0197-y)

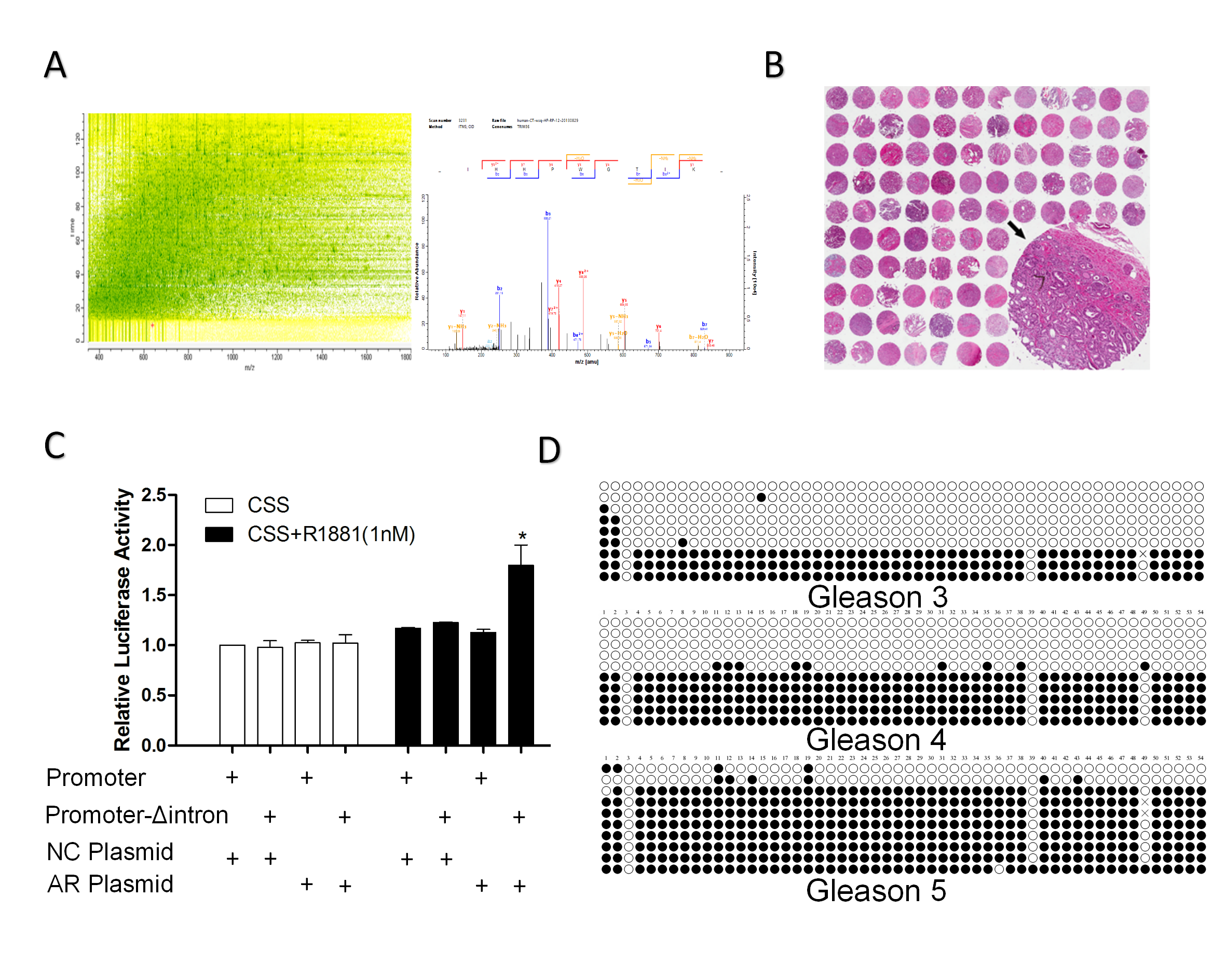

Supplement: Supplementary file 1 — Figure S1 [file 41419_2017_197_MOESM1_ESM.tif]
