# Supplementary material for: TRIM36, a novel androgen-responsive gene, enhances anti-androgen efficacy against prostate cancer by inhibiting MAPK/ERK signaling pathways
Source: Cell Death Dis. 2018 Feb 5;9(2):155. doi: 10.1038/s41419-017-0197-y (PMC5833828; doi:10.1038/s41419-017-0197-y)
Supplement: Supplementary file 2 — Table S1 [file 41419_2017_197_MOESM2_ESM.doc]

Table.S1 Characteristics of the study population of 95 patients treated by radical prostatectomy.

| Age |  |  |
| --- | --- | --- |
| Mean±SD(year) | 69.3±5.99 |  |
| <60 | 6 | 6.32% |
| 60-70 | 42 | 44.21% |
| >70 | 47 | 49.47% |
| Pre-operative PSA(ng/ml) |  |  |
| <10 | 27 | 28.42% |
| 10-20 | 30 | 31.58% |
| >20 | 38 | 40.00% |
| Gleason score |  |  |
| =3+4or ≤6 | 56 | 58.95% |
| =4+3 or ≥8 | 39 | 41.05% |
| Pathological stage |  |  |
| pT2 | 80 | 84.21% |
| pT3/ T4 | 15 | 15.79% |
| Time to PSA progression |  |  |
| Mean ± SD (months) | 26.2±19.0 |  |
| Overall follow-up |  |  |
| Mean ± SD (months) | 48.7±26.6 |  |

SD, standard deviation
